# Connecting the Dots: From DNA Damage and Repair to Aging

**DOI:** 10.3390/ijms17050685

**Published:** 2016-05-06

**Authors:** Mei-Ren Pan, Kaiyi Li, Shiaw-Yih Lin, Wen-Chun Hung

**Affiliations:** 1 Graduate Institute of Clinical Medicine, College of Medicine, Kaohsoung Medical University, Kaohsiung 807, Taiwan; mrpan@cc.kmu.edu.tw; 2 The Michael E. DeBakey Department of Surgery, Baylor College of Medicine, Houston, TX 77030, USA; kli@bcm.edu; 3 Department of Systems Biology, MD Anderson Cancer Center, Houston, TX 77030, USA; sylin@mdanderson.org; 4 National Institute of Cancer Research, National Health Research Institutes, Tainan 704, Taiwan

**Keywords:** DNA damage response, senescence, aging

## Abstract

Mammalian cells evolve a delicate system, the DNA damage response (DDR) pathway, to monitor genomic integrity and to prevent the damage from both endogenous end exogenous insults. Emerging evidence suggests that aberrant DDR and deficient DNA repair are strongly associated with cancer and aging. Our understanding of the core program of DDR has made tremendous progress in the past two decades. However, the long list of the molecules involved in the DDR and DNA repair continues to grow and the roles of the new “dots” are under intensive investigation. Here, we review the connection between DDR and DNA repair and aging and discuss the potential mechanisms by which deficient DNA repair triggers systemic effects to promote physiological or pathological aging.

## 1. Introduction

We witnessed a fruitful DNA damage response and repair year in 2015. Three pioneers in the study of DNA repair systems, Tomas Lindahl, Paul Modrich, and Aziz Sancar, received the Nobel Prize in Chemistry which recognizes their contribution in the finding and elucidation of base excision repair, mismatch repair and nucleotide excision repair, respectively [1]. At the same time, the Albert Lasker Basic Medical Research Award honors Evelyn M. Witkin and Stephen J. Elledge for their discoveries of a critical genome protection mechanism, the DNA damage response (DDR) [2].

DNA carries the inheritable genetic information for all living organisms. However, DNA receives endogenous and exogenous insults every minute and the lesions (approximately 10^4^–10^5^ per cell per day) are extremely deleterious to cells [3]. These lesions, if not correctly repaired, will interrupt genome replication and transcription and cause wide-scale chromosomal aberrations that trigger malignant transformation or cell death. Therefore, effective sensing and repair systems are developed during evolution to eliminate the DNA lesions and to maintain genome integrity. Dysregulation of DDR and repair is closely associated with human diseases such as cancers, cardiovascular disease, neurodegenerative disorders and aging [4,5,6,7].

## 2. The DDR Signaling Cascade

DNA damage is caused by a wide variety of environmental agents, genotoxic chemicals, and cellular metabolism. Depending on the source of damage, DNA can be altered in different ways, including nucleotide alterations (mutation, substitution, deletion and insertion), bulky adducts, single-strand breaks (SSB) and double-strand breaks [8]. To guide accurate repair, cells elicit a highly specific response network to detect the damage sites with sensing molecules (sensors) and then transmit the damage signals to the transducers which are composed of a number of protein kinases. Finally, different checkpoints and repair systems (effectors) including cell cycle regulators, nucleases, helicases, polymerases, ligases, *etc.*, are engaged in the execution of the repair of damage to preserve genomic integrity. The detailed signaling cascade and repair mechanism have been discussed in detail [9,10,11]. Here, we only provide a brief introduction to DDR signaling and DNA repair.

Currently, four repair mechanisms for damaged DNA have been elucidated in mammalian cells. Base excision repair (BER) mainly corrects single lesions or small alternations of bases. Nucleotide excision repair (NER) is a more complex process involving the removal of bulky DNA lesions. Homologous recombination (HR) and non-homologous end joining (NHEJ) mainly work on double-strand break (DSB) repair. Different types of DNA damage could be fixed by specific repair mechanisms. In BER/SSB repair, the poly(ADP-ribose) polymerase protein 1 (PARP1) is activated and then recognizes the DNA lesion by the zinc-binding domain. Subsequently, PARP1 assembles poly(ADP)-ribose (PAR) chains on target proteins, including histone H1, H2B and PARP1 itself, and the PAR structures act as platforms to recruit multiple protein complexes which function in DNA repair and chromatin modification. DDR proteins such as XRCC1 and LIG3 which contain an acidic-basic residue-rich motif can read the PAR structures and are recruited to the DNA damage sites to initiate the repair process [12,13]. The initial activation of DDR by DSB underlies the protein kinase cascades. Ataxia telangiectasia, mutated (ATM), ATM- and Rad3-related (ATR), and DNA-dependent protein kinase (DNA-PK) are regarded as sensors of DNA damage [14,15,16]. Once DNA damage occurs, H2AX is rapidly phosphorylated at Ser139 (γ-H2AX) at DSBs by ATM, ATR and DNA-PKcs [17,18,19]. Then γ-H2AX decorates the 30 kb region of chromatin-flanking DSBs and recruits early DDR proteins to the damaged sites to generate foci formation and to initiate the repair process [20]. Thus, the spatiotemporal recruitment of sensors, transducers and effectors orchestrates a tightly controlled process to repair each type of DNA damage.

However, DDR functions as “a double-edged sword” in controlling cell survival. Although DDR acts as a native guardian of genomic stability to prevent cell transformation, cancer cells with high DDR tend to develop resistance to chemotherapy and radiotherapy. Therefore, the balance of DNA damage and DDR is highly relevant to both cancer development and effective chemotherapy. The relation between impaired DDR and human diseases is discussed in the following.

## 3. The DDR and Human Diseases

### 3.1. Cancer and DNA Damage

It has been well known that aberrant oncogene activation or inactivation of certain tumor-suppressor genes are fundamental features in cancers. Notably, evidence shows that mutations in oncogenes and tumor suppressors in the precancerous lesions not only display a high proliferation rate, but also exhibit persistent DNA damage, known as replication stress [21]. The common features of replication stress are a low level of DNA synthesis, increased numbers of stalled and collapsed replication forks that result from deregulated replication origins, elongating replication fork, post-replicative DNA repair and S phase checkpoint and, subsequently, accumulated DNA damage. For example, cyclin E alters the licensing and firing of the replication origin that leads to ssDNA and elevates replication stress in cells [22]. Reduced binding of replicative helicase minichromosome maintenance complex 2-7 (MCM2-7) at replication origins also induced replication stress and genome instability [23]. Failures of DNA helicase–mediated replication fork resumption are known to be associated with cancer predisposition [24,25,26,27]. The replication stress response (also called intra-S phase checkpoint) acts as a natural barrier against tumorigenesis at the premalignant stage. Once the replication stress response becomes defective, normal cells may transform into cancer cells. ATR and CHK1 kinase are key mediators for the response to replication stress in triggering cell cycle arrest and replication fork stabilization and restart. Loss of the *ATR* gene leads to chromosomal fragmentation and early embryonic lethality [28]. The BRCA protein is another example that links HR repair proteins, replication stress and cancer. It has been known that BRCA proteins promote error-free HR repairs that function as the blockage of error-prone NHEJ pathways [29,30]. The mutation of *Brca1* in mammary epithelial cells results in tumor formation [31]. Therefore, the DDR-like replication stress response plays an important tumor-suppressive role in leading cell cycle arrest or apoptosis to maintain genome integrity. Consistent with *in vitro* studies, oncogene-induced replication stress has also been demonstrated in *in vivo* mouse cancer models and human tumors [32,33]. This evidence suggested that replication stress–induced DDR in pre-cancerous and cancerous cells plays a vital role in tumorigenesis.

### 3.2. Cardiovascular Diseases and DNA Damage

Recently, growing evidence demonstrated that cells with high levels of oxidative stress and abnormalities in DNA repair pathways are found in heart failure patients [34,35]. Oxidative stress induced by diverse stimuli including angiotensin II, oxidized low density lipoproteins, and conditional stretch high shear stress causes serious injuries in cardiac myocytes and vascular endothelial cells. These cells exhibit lower DNA repair activity, and therefore are highly sensitive to oxidative stress. Reactive oxygen species (ROS) widely causes the production of catastrophic DNA damage in heart failure patients with vascular endothelial dysfunction, cardiac hypertrophy and myocyte dysfunction. It is suggested that prevention of oxidative stress–associated DNA damage or enhancement of DNA repair activity may represent a novel therapeutic strategy in cardiovascular diseases.

### 3.3. Neurodegenerative Disorders and DNA Damage

DNA repair is extremely important in the early developmental stages of the nervous system, because unrepaired lesions can cause hazardous effects on the function of nervous system. Studies using human samples and animal models have demonstrated a close relation between abnormal DDR and neurodegeneration [36,37,38]. Xeroderma pigmentosum (XP) patients exhibited several neurological symptoms such as microcephaly, mental retardation and deafness, which are linked to mutations in genes such as *XPA*, *XPB*, *XPD*, *XPF*, and *XPG* that are involved in nucleotide excision repair (NER) [39]. In addition to XP, numerous congenital mutations in the components in NER, SSB and DSB repair result in neurodegenerative syndromes such as Cockayne syndrome (CS), trichothiodystrophy (TTD) and Ataxia Telangiectasia (AT) [40]. Moreover, insufficient DNA repair also plays a role in Alzheimer’s, Huntington’s and Parkinson’s diseases [41,42]. This evidence strongly supports the possibility that a defective DDR contributes significantly to neurodegenerative disorders.

### 3.4. Aging and DNA Damage

Aging is a universally conserved feature among eukaryotic organisms. It is characterized by a progressive decline of physiological integrity in molecules, cells, tissues and organisms. Age-related pathological changes include atherosclerosis, heart failure, renal failure, neurodegeneration, osteoporosis, *etc.*, in mammals. Our understanding of the molecular basis of aging processes will be helpful for the prevention or treatment of age-related diseases. Recent results from anti-aging studies provide interesting crosstalk between DDR and aging. The depletion of mitochondria-associated senescence genes or mTORC1 inhibition can efficiently block aging phenotypes [43,44]. The decrease of Complex II activity in mitochondria with age is found in senescent skin fibroblasts [45]. Recently, nine hallmarks of aging including genome instability, telomere attrition, epigenetic alterations, cellular senescence, mitochondrial dysfunction, loss of proteostasis, deregulated nutrient sensing, stem cell exhaustion, and altered intercellular communication have been enumerated to represent common features in mammalian cells [46]. Because one of the common origins of aging is the accumulation of damaged DNA throughout life, we attempt to connect DNA damage and repair to aging in this review.

## 4. The Linkage between DNA Damage/Repair and Aging

Aging is defined as a progressive decline of body function and a decrease of physiological response to stress that ultimately results in death. Because the insufficiency of repair will cause the accumulation of DNA damage which leads to cell death or functional defect, it is reasonable to hypothesize that DDR and repair is closely associated with aging [47]. Indeed, mice defective in DNA repair exhibit features of premature aging. For example, trichothiodystrophy (TTD) mice with a mutation in *XPD*, which encodes a DNA helicase in DNA repair, have a short lifespan and develop aging symptoms such as osteoporosis and cachexia at a young stage [48]. Human genetic diseases with DNA repair defects such as Huchinson-Gilford Progeria, CS, Werner sundrome, AT, Nijmegen breakage syndrome, Fanconi anemia and Bloom syndrome all show premature aging. However, not all DNA damage and repair cause aging. Defects in mismatch repair (MMR) may result in cancer formation but not directly correlate with aging [49]. In contrast to MMR, deficiencies in BER, NER, NHEJ and HR indeed induce aging-associated phenotypes. For example, mitochondrial BER (mtBER) completes DNA repair in oxidative lesions via four distinct enzymes including DNA glycosylase, endonuclease, DNA polymerase γ and DNA ligase to remove the damaged base. Reduction of the activity of these enzymes has been shown to promote aging [50,51,52]. Similarly, Werner syndrome patients with an inherited defect in BER and CS and XP patients with a deficiency in NER all show features of premature aging. In addition, NHEJ is significantly reduced in the neurons from old rats and a decrease in Ku70, Ku80 and Mre11 is also frequently found in aged animals [53,54]. Thus, an increase of DNA damage and mutation or a decrease of DNA repair is linked with age-associated diseases. A list of syndromes carrying defects in DNA repair is shown in Table 1.

## 5. Senescence and Aging

As previously mentioned, an accumulation of DNA damage or defect in DNA repair promotes the aging process. Interestingly, an accumulation of DNA damage or defect in DNA repair also promotes cellular senescence and apoptosis. This raises the question whether senescence induced by physiological or pathological alterations may be involved in aging. Therefore, we further discuss the correlation between senescence and aging in cells, animals and human diseases.

### 5.1. Evidence from Cellular Study

Senescence is a cellular phenotype first described by Hayflick and Moorhead in 1961 [55]. They demonstrated that fibroblasts stayed in permanent growth arrest with an enlarged cell size after serial cultivation. Subsequent studies revealed two major types of senescence, replicative senescence which results from the progressive shorting of telomeres at the end of chromosome DNA after continuous replication, and stress-induced senescence triggered by the overexpression of oncogenes, oxidative stress, ultraviolet (UV), *etc.*, to promote senescence [56,57]. Previous studies showed that oncogene-induced senescence in fibroblasts is a DDR triggered by replication dysregulation, because of the elevated replication stress from unregulated proliferation [58,59]. Several pioneer studies further demonstrated that DNA damage triggers p53-dependent senescence in normal human fibroblasts, providing a strong linkage between DDR and senescence [60]. *p16^Ink^*^4*a*^ is another senescence master regulator. Life-long removal of *p16^Ink^*^4*a*^ can delay the aging process [61]. These results suggested that DDR activated by replicative- or stress-induced senescence plays a causative role in promoting cellular aging.

### 5.2. Evidence from Animal Models

Based on the evidence of cellular senescence, intensive investigations have been carried out to address the role of DDR proteins in aging. DNA repair–deficient nematodes have a significantly shorter life span while several long-living mutants show increased repair activity, suggesting DNA repair capacity influences the aging process and affects longevity in nematodes [62]. Results of genetically modified mice studies also support a preventive function of DDR proteins on aging. Wong *et al.* demonstrated that telomere dysfunction and Atm deficiency accelerates the aging process in mice [63]. Similarly, the absence of breast cancer 1 (Brca1) full-length isoform causes senescence in embryos and aging in adult mice [64]. Deletion of Atr in adult mice also leads to age-related phenotypes and stem cell loss [65]. In addition to protein kinases, knockout of Ku80, a non-homologous end joining protein involved in DSB repair, showed early aging in mice [66]. These data suggested that deficiency in DNA repair promotes aging *in vivo*.

### 5.3. Evidence from Human Diseases

As discussed above, a number of human genetic defects including CS and XP also suggest that deficient DNA repair leads to tissue degeneration and premature aging. CS is an autosomal recessive disease mainly caused by mutations in Cockayne syndrome group A (CSA) (also known as excision repair cross-complementation group 8, ERCC8) and Cockayne syndrome group B (CSB) (also known as ERCC6) genes. Both genes participate in the excision repair pathways [67]. CS patients show impairment of the nervous system, hypersensitivity to sunlight, retinal disorder and premature aging [68,69]. Similarly, XP patients have photosensitivity of the skin and eye and exhibit premature cutaneous aging with increased incidence of basal cell carcinoma and melanoma [70,71]. The characteristics of the CS and XP patients also suggest that DNA repair is closely associated with aging.

## 6. From Single Cell to Systemic Effect

**Figure 1:**
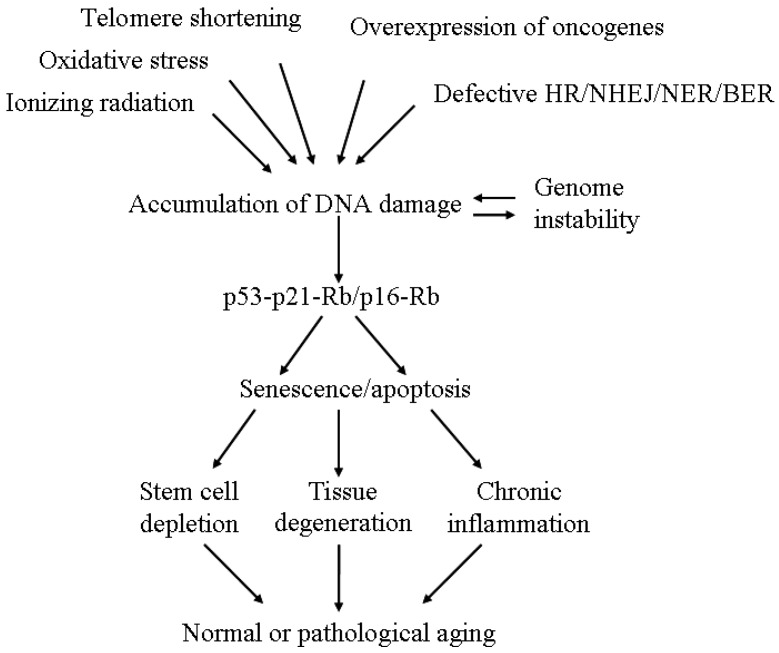
A model for the role of unresolved DNA lesions in aging.

Many previous studies addressing the senescence mechanism were done in single cells, especially in fibroblasts. An obvious question is how cellular senescence caused by deficient DNA repair finally affects the aging of a living organism. We propose three potential mechanisms to explain the systemic effect. First, senescence depletes the supplemental pool of stem cells or progenitor cells that leads to the continuous decline of tissue homeostasis and accelerates organ aging. An elegant study from DePinho’s laboratory demonstrated that mice lacking telomerase RNA showed genomic instability in cells and functional defects in multiple highly proliferative organs [72]. Although animal aging was not described in the study, it is predictable that these mice will have a short lifespan due to impaired tissue renewal. Readers are suggested to refer to a recent review article for the functional role of stem cells in aging [73]; Secondly, senescence causes tissue degeneration. As evidenced in human diseases, defects in DNA repair induce senescence and degeneration of nervous and endocrine/exocrine tissues. Dysfunction of the nervous system would decrease the activity of innervated tissues and dysfunction of the endocrine/exocrine system would disturb hormone homeostasis and nutrient balance which ultimately causes organ aging; Thirdly, senescence induces chronic inflammation. One well-known characteristic of senescent cells is the production of pro-inflammatory and matrix-degrading molecules, known as the senescence-associated secretory phenotype (SASP) [74,75]. Higher serum levels of pro-inflammatory factors such as interleukin-6 and tumor necrosis factor are found in aged mice [76,77]. A similar observation is also confirmed in aged individuals [78]. Chronic inflammation triggered by these pro-inflammatory factors changes the immune response and vascular system and finally disrupts the physiological function of many tissues to promote the aging process [79]. Figure 1 shows the hypothesis by which DNA repair–deficient senescence promotes aging.

## 7. New Dots Connecting DNA Damage and Repair to Aging

More than 50 proteins are recruited to the lesion sites after DNA damage occurs [80]. These proteins quickly undergo post-translational modifications including phosphorylation, sumoylation, ubiquitination, *etc.*, to modulate the protein-protein interaction and to fine-tune the enzymatic activity to repair DNA. It is not surprising that in addition to the core DDR kinases, many accessory proteins also affect the DNA repair and aging process. In addition, recent studies also point out the importance of mitochondria function in DNA damage and aging. We discuss these “new dots” in the DDR and their potential role in aging.

### 7.1. Ubiquitin-Specific Protease 3 (USP3)

Ubiquitination of the histone protein at DNA breaks is an essential step for the activation of DDR and DNA repair. The addition of ubiquitin to histones is kinetically regulated by ubiquitin ligases and deubiquitinating enzymes. USP3 is a deubiquitinating enzyme for monoubiquitinated histone H2A and H2B [81]. A previous *in vitro* study demonstrated a role of this enzyme in the activation of DDR because depletion of this protein leads to a delay of S phase progression and the accumulation of DNA breaks. Recently, Lancini *et al.* generated USP3 knockout mice and found that deletion of this gene caused a defective DDR and chromosomal instability [82]. In addition, these mice have a reduced hematopoietic stem cell reservoir and shortened life span. This study provides the *in vivo* evidence to confirm the role of USPs in aging.

### 7.2. N-Terminal RCC1 Methyltransferase 1 (NRMT1)

NRMT1 is the first identified eukaryotic α-*N*-methyltransferase which methylates a three-amino-acid *N*-terminal consensus sequence [83]. A bioinformatics search predicts over 300 potential substrates for this enzyme and some of them are involved in DNA repair. Subsequent studies confirmed a critical function of NRMT1 in NER [84]. Recently, Bonsignore *et al.* demonstrated that NRMT1 knockout mice exhibit decreased body size, female-specific infertility, reduced mitochondria function and early-onset liver degeneration [85]. In addition, these mice showed premature aging similar to the phenotypes found in other mouse models with defects in DNA repair. Therefore, NRMT1 is a new player in NER that is also implicated in aging.

### 7.3. Mitochondria Dysfunction in XP and CS

Previous studies on DDR and repair focus on genomic DNA. However, two recent studies elucidate a new role of mitochondria in DDR and aging. Fang *et al.* found that *XPA*–deficient cells exhibited hyperactivation of PARP-1 and decreased activation of the NAD^+^/SIRT1/PGC-1α signaling which results in defective mitophagy and mitochondria dysfunction [86]. Inhibition of PARP-1 or supplementation of NAD^+^ precursors rescued the lifespan in xpa-1 nematodes. These results imply mitochondria in XP-induced premature aging. In another study, Scheibye-Knudsen *et al.* also found aberrant PARP activation, decreased SIRT1 activity and mitochondria dysfunction in CS mice and human CS cells [87]. Similarly, stimulation of SIRT1 activity by NAD^+^ supplementation rescues CS-associated phenotypes including accelerated aging.

### 7.4. Nonenzymatic Post-Translational Modification

The aging process is known to be associated with increased oxidative stress, which induces post-translational modifications of proteins including glycation, glycoxidation, lipoxidation and carbonylation. Recently, it has been reported that proteins tend to become more insoluble and aggregate during physiological aging due to these modification [88]. A study further indicated that accumulation of extracellular advanced glycation end products (AGEs) in Alzheimer’s disease (AD) is caused by an accelerated oxidation of glycated proteins (“glycoxidation”). These observations support that AGEs are involved in the pathogenesis of AD [89,90].

### 7.5. RecQ-Like Helicase Sgs1

Sgs1 is a RecQ-like helicase identified in budding yeast and it plays an important role in the repair of DSB by resecting DSB ends in collaboration with nucleases [91]. Sgs1 defects in humans are associated with premature aging syndromes. Recently, it was found that small ubiquitin-like modifier (SUMO)-targeted ubiquitin ligase (STUbL) complex Slx5–Slx8 negative regulated Sgs1 foci formation under DNA damage [92]. Therefore, this result provides another model to address ubiquitination being involved in aging formation.

## 8. Conclusions

Our understanding of DDR and DNA repair has tremendously advanced in the past two decades. In addition, the role of DDR and repair in cancer and normal or pathological aging has become much clearer. However, the translation of the knowledge into clinical application is still at a very early stage. Currently, only one PARP inhibitor, Olaparib (Lymparza, AstraZeneca, Esbo, Finland), has been approved by the FDA for cancer treatment and many challenges remained in the development or clinical utility of PARP inhibitors. For aging or age-related diseases, the condition is more complex because DDR and DNA damage–triggered senescence could be a barrier for tumor formation. With the appearance of “new dots” in the signaling network, we may have the opportunity to develop new strategies to deal with cancer and aging in the future.

## Figures and Tables

**Table 1:** A list of age-associated diseases carrying defects in genome maintenance.

Disease	Mutated Genes	Repair Pathways Affected
Breast cancer; ovarian cancer	*BRCA1*; *BRCA2*; *MRE11*	HR
Ataxia telangiectasia	*ATM*	DSB repair
Nijmegen breakage syndrome	*NBS1*	DSB repair; telomere maintenance
Bloom syndrome	*BLM*	Mitotic recombination
Fanconi anemia	*FANC*; *BRCA2*	DNA crosslink repair
Breast cancer; sarcoma; brain cancer; adenocotical carcinoma	*P53*	HR; BER; NER; NHEJ
Cockayne syndrome	*CSA*; *CSB*	TC-NER; GG-NER
Trichothiodystrophy	*XPB*; *XPD*; *XPG*	TC-NER; GG-NER
Hutchison-Gilford progeria syndrome	*LMNA*	Nuclear lamina function
Xeroderma pigmentosum	*XPC*	GG-NER
Werner syndrome	*WRN*	telomere maintenance; DNA recombination repair

Abbreviations: TC-NER: transcription-couple nucleotide excision repair; GG-NER: global-genome nucleotide excision repair.

## References

[1] Kunkel T.A. (2015). Celebrating DNA’s repair crew. Cell.

[2] Haber J.E. (2015). Deciphering the DNA damage response. Cell.

[3] Hoeijmakers J.H. (2009). DNA damage, aging, and cancer. N. Engl. J. Med..

[4] Madabhushi R., Pan L., Tsai L.H. (2014). DNA damage and its links to neurodegeneration. Neuron.

[5] Ishida T., Ishida M., Tashiro S., Yoshizumi M., Kihara Y. (2014). Role of DNA damage in cardiovascular disease. Circ. J..

[6] Chow H.M., Herrup K. (2015). Genomic integrity and the ageing brain. Nat. Rev. Neurosci..

[7] Dobbelstein M., Sorensen C.S. (2015). Exploiting replicative stress to treat cancer. Nat. Rev. Drug Discov..

[8] Rodriguez-Rocha H., Garcia-Garcia A., Panayiotidis M.I., Franco R. (2011). DNA damage and autophagy. Mutat. Res..

[9] Zhou B.B., Elledge S.J. (2000). The DNA damage response: Putting checkpoints in perspective. Nature.

[10] Ciccia A., Elledge S.J. (2010). The DNA damage response: Making it safe to play with knives. Mol. Cell.

[11] Seviour E.G., Lin S.Y. (2010). The DNA damage response: Balancing the scale between cancer and ageing. Aging.

[12] Ménissier-de Murcia J., Molinete M., Gradwohl G., Simonin F., de Murcia G. (1989). Zinc-binding domain of poly(ADP-ribose)polymerase participates in the recognition of singlestrand breaks on DNA. J. Mol. Biol..

[13] Caldecott K.W. (2014). Protein ADP-ribosylation and the cellular response to DNA strand breaks. DNA Repair.

[14] Rogakou E.P., Boon C., Redon C., Bonner W.M. (1999). Megabase chromatin domains involved in DNA double-strand breaks *in vivo*. J. Cell Biol..

[15] Harper J.W., Elledge S.J. (2007). The DNA damage response: Ten years after. Mol. Cell.

[16] Jackson S.P., Bartek J. (2009). The DNA-damage response in human biology and disease. Nature.

[17] Burma S., Chen B.P., Murphy M., Kurimasa A., Chen D.J. (2001). ATM Phosphorylates histone H2AX in response to DNA double-strand breaks. J. Biol. Chem..

[18] Podhorecka M., Skladanowski A., Bozko P. (2010). H2AX phosphorylation: Its role in DNA damage response and cancer therapy. J. Nucleic Acids.

[19] Stiff T., O’Driscoll M., Rief N., Iwabuchi K., Lobrich M., Jeggo P.A. (2004). ATM and DNA-PK function redundantly to phosphorylate H2AX after exposure to ionizing radiation. Cancer Res..

[20] Shroff R., Arbel-Eden A., Pilch D., Ira G., Bonner W.M., Petrini J.H., Haber J.E., Lichten M. (2004). Distribution and dynamics of chromatin modification induced by a defined DNA double-strand break. Curr. Biol..

[21] Gaillard H., Garcia-Muse T., Aguilera A. (2015). Replication stress and cancer. Nat. Rev. Cancer.

[22] Jones R.M., Mortusewicz O., Afzal I., Lorvellec M., Garcia P., Helleday T., Petermann E. (2013). Increased replication initiation and conflicts with transcription underlie cyclin e-induced replication stress. Oncogene.

[23] Shima N., Alcaraz A., Liachko I., Buske T.R., Andrews C.A., Munroe R.J., Hartford S.A., Tye B.K., Schimenti J.C. (2007). A viable allele of Mcm4 Causes chromosome instability and mammary adenocarcinomas in mice. Nat. Genet..

[24] Brosh R.M. (2013). DNA Helicases involved in DNA repair and their roles in cancer. Nat. Rev. Cancer.

[25] Hu Y., Raynard S., Sehorn M.G., Lu X., Bussen W., Zheng L., Stark J.M., Barnes E.L., Chi P., Janscak P. (2007). RECQL5/Recql5 helicase regulates homologous recombination and suppresses tumor formation via disruption of Rad51 presynaptic filaments. Genes Dev..

[26] Mann M.B., Hodges C.A., Barnes E., Vogel H., Hassold T.J., Luo G. (2005). Defective sister-chromatid cohesion, aneuploidy and cancer predisposition in a mouse model of type II rothmund-thomson syndrome. Hum. Mol. Genet..

[27] Luo G., Santoro I.M., McDaniel L.D., Nishijima I., Mills M., Youssoufian H., Vogel H., Schultz R.A., Bradley A. (2000). Cancer predisposition caused by elevated mitotic recombination in bloom mice. Nat. Genet..

[28] Gilad O., Nabet B.Y., Ragland R.L., Schoppy D.W., Smith K.D., Durham A.C., Brown E.J. (2010). Combining ATR suppression with oncogenic Ras synergistically increases genomic instability, causing synthetic lethality or tumorigenesis in a dosage-dependent manner. Cancer Res..

[29] Willis N.A., Chandramouly G., Huang B., Kwok A., Follonier C., Deng C., Scully R. (2014). BRCA1 controls homologous recombination at Tus/Ter-stalled mammalian replication forks. Nature.

[30] Hakem R., de la Pompa J.L., Sirard C., Mo R., Woo M., Hakem A., Wakeham A., Potter J., Reitmair A., Billia F. (1996). The tumor suppressor gene *Brca1* is required for embryonic cellular proliferation in the mouse. Cell.

[31] Xu X., Wagner K.U., Larson D., Weaver Z., Li C., Ried T., Hennighausen L., Wynshaw-Boris A., Deng C.X. (1999). Conditional mutation of Brca1 in mammary epithelial cells results in blunted ductal morphogenesis and tumour formation. Nat. Genet..

[32] Murga M., Bunting S., Montana M.F., Soria R., Mulero F., Canamero M., Lee Y., McKinnon P.J., Nussenzweig A., Fernandez-Capetillo O. (2009). A mouse model of ATR-seckel shows embryonic replicative stress and accelerated aging. Nat. Genet..

[33] Zeman M.K., Cimprich K.A. (2014). Causes and consequences of replication stress. Nat. Cell Biol..

[34] Tsutsui H., Kinugawa S., Matsushima S. (2011). Oxidative stress and heart failure. Am. J. Physiol. Heart Circ. Physiol..

[35] Giordano F.J. (2005). Oxygen, oxidative stress, hypoxia, and heart failure. J. Clin. Investig..

[36] De B.J., Donker I., de W.J., Hoeijmakers J.H., Weeda G. (1998). Disruption of the mouse xeroderma pigmentosum group D DNA repair/basal transcription gene results in preimplantation lethality. Cancer Res..

[37] De B.J., de W.J., van S.H., Berg R.J., Morreau H., Visser P., Lehmann A.R., Duran M., Hoeijmakers J.H., Weeda G. (1998). A mouse model for the basal transcription/DNA repair syndrome trichothiodystrophy. Mol. Cell.

[38] Barlow C., Hirotsune S., Paylor R., Liyanage M., Eckhaus M., Collins F., Shiloh Y., Crawley J.N., Ried T., Tagle D. (1996). Atm-deficient mice: A paradigm of ataxia telangiectasia. Cell.

[39] Iyama T., Wilson D.M. (2013). DNA Repair mechanisms in dividing and non-dividing cells. DNA Repair.

[40] Laposa R.R., Huang E.J., Cleaver J.E. (2007). Increased apoptosis, P53 up-regulation, and cerebellar neuronal degeneration in repair-deficient cockayne syndrome mice. Proc. Natl. Acad. Sci. USA.

[41] Shokolenko I., Venediktova N., Bochkareva A., Wilson G.L., Alexeyev M.F. (2009). Oxidative stress induces degradation of mitochondrial DNA. Nucleic Acids Res..

[42] Tuppen H.A., Blakely E.L., Turnbull D.M., Taylor R.W. (2010). Mitochondrial DNA mutations and human disease. Biochim. Biophys. Acta.

[43] Johnson S.C., Rabinovitch P.S., Kaeberlein M. (2013). mTOR is a key modulator of ageing and age-related disease. Nature.

[44] Correia-Melo C., Marques F.D., Anderson R., Hewitt G., Hewitt R., Cole J., Carroll B.M., Miwa S., Birch J., Merz A. (2016). Mitochondria are required for pro-ageing features of the senescent phenotype. EMBO J..

[45] Bowman A., Birch-Machin M.A. (2016). Age-dependent decrease of mitochondrial complex II activity in human skin fibroblasts. J. Investig. Dermatol..

[46] Lopez-Otin C., Blasco M.A., Partridge L., Serrano M., Kroemer G. (2013). The hallmarks of aging. Cell.

[47] Lombard D.B., Chua K.F., Mostoslavsky R., Franco S., Gostissa M., Alt F.W. (2005). DNA repair, genome stability, and aging. Cell.

[48] De B.J., Andressoo J.O., de W.J., Huijmans J., Beems R.B., van Steeq H., Weeda G., van der Horst G.T., van Leeuwen W., Themmen A.P. (2002). Premature aging in mice deficient in DNA repair and transcription. Science.

[49] Papadopoulos N., Lindblom A. (1997). Molecular basis of HNPCC: Mutations of MMR genes. Hum. Mutat..

[50] Boesch P., Weber-Lotfi F., Ibrahim N., Tarasenko V., Cosset A., Paulus F., Lightowlers R.N., Dietrich A. (2011). DNA repair in organelles: Pathways, organization, regulation, relevance in disease and aging. Biochim. Biophys. Acta.

[51] Maynard S., Schurman S.H., Harboe C., de Souza-Pinto N.C., Bohr V.A. (2009). Base excision repair of oxidative DNA damage and association with cancer and aging. Carcinogenesis.

[52] Gorbunova V., Seluanov A., Mao Z., Hine C. (2007). Changes in DNA repair during aging. Nucleic Acids Res..

[53] Vyjayanti V.N., Rao K.S. (2006). DNA double strand break repair in brain: Reduced NHEJ activity in aging rat neurons. Neurosci. Lett..

[54] Li H., Vogel H., Holcomb V.B., Gu Y., Hasty P. (2007). Deletion of Ku70, Ku80, or both causes early aging without substantially increased cancer. Mol. Cell. Biol..

[55] Hayflick L., Moorhead P.S. (1961). The serial cultivation of human diploid strains. Exp. Cell Res..

[56] Campisi J. (2005). Senescent cells, tumor suppression, and organismal aging: Good citizens, and bad neighbors. Cell.

[57] Courtois-Cox S., Jones S.L., Cichowski K. (2008). Many roads lead to oncogene-induced senescence. Oncogene.

[58] Di Micco R., Fumagalli M., Cicalese A., Piccinin S., Gasparini P., Luise C., Schurra C., Garre’ M., Nuciforo P.G., Bensimon A. (2006). Oncogene-induced senescence is a DNA damage response triggered by DNA hyper-replication. Nature.

[59] Bartkova J., Rezaei N., Liontos M., Karakaidos P., Kletsas D., Issaeva N., Vassiliou L.V., Kolettas E., Niforou K., Zoumpourlis V.C. (2006). Oncogene-induced senescence is part of the tumorigenesis barrier imposed by DNA damage checkpoints. Nature.

[60] Tyner S.D., Venkatachalam S., Choi J., Jones S., Ghebranious N., Igelmann H., Lu X., Soron G., Cooper B., Brayton C. (2002). P53 mutant mice that display early ageing-associated phenotypes. Nature.

[61] Baker D.J., Wijshake T., Tchkonia T., LeBrasseur N.K., Childs B.G., van de Sluis B., Kirkland J.L., van Deursen J.M. (2011). Clearance of P16Ink4a-positive senescent cells delays ageing-associated disorders. Nature.

[62] Hyun M., Lee J., Lee K., May A., Bohr V.A., Ahn B. (2008). Longevity and resistance to stress correlate with DNA repair capacity in *Caenorhabditis elegans*. Nucleic Acids Res..

[63] Wong K.K., Maser R.S., Bachoo R.M., Menon J., Carrasco D.R., Gu Y., Alt F.W., DePinho R.A. (2003). Telomere dysfunction and Atm deficiency compromises organ homeostasis and accelerates ageing. Nature.

[64] Cao L., Li W., Kim S., Brodie S.G., Deng C.X. (2003). Senescence, aging, and malignant transformation mediated by p53 in mice lacking the Brca1 full-length isoform. Genes Dev..

[65] Ruzankina Y., Pinzon-Guzman C., Asare A., Ong T., Pontano L., Cotsarelis G., Zediak V.P., Velez M., Bhandoola A., Brown E.J. (2007). Deletion of the developmentally essential gene ATR in adult mice leads to age-related phenotypes and stem cell loss. Cell Stem Cell.

[66] Holcomb V.B., Vogel H., Hasty P. (2007). Deletion of Ku80 causes early aging independent of chronic inflammation and Rag-1-induced DSBs. Mech. Ageing Dev..

[67] Lindahl T., Karran P., Wood R.D. (1997). DNA excision repair pathways. Curr. Opin. Genet. Dev..

[68] Bertola D.R., Cao H., Albano L.M., Oliveira D.P., Kok F., Marques-Dias M.J., Kim C.A., Hegele R.A. (2006). Cockayne syndrome type A: Novel mutations in eight typical patients. J. Hum. Genet..

[69] Bender M.M., Potocki L., Metry D.W. (2003). What syndrome is this? Cockayne syndrome. Pediatr. Dermatol..

[70] Robbins J.H., Kraemer K.H., Lutzner M.A., Festoff B.W., Coon H.G. (1974). Xeroderma pigmentosum. An inherited diseases with sun sensitivity, multiple cutaneous neoplasms, and abnormal DNA repair. Ann. Intern. Med..

[71] Robbins J.H. (1988). Xeroderma pigmentosum. Defective DNA repair causes skin cancer and neurodegeneration. Jpn. Automob. Manuf. Assoc..

[72] Lee H.W., Blasco M.A., Gottlieb G.J., Horner J.W., Greider C.W., DePinho R.A. (1998). Essential role of mouse telomerase in highly proliferative organs. Nature.

[73] Goodell M.A., Rando T.A. (2015). Stem cells and healthy aging. Science.

[74] Childs B.G., Durik M., Baker D.J., van Deursen J.M. (2015). Cellular senescence in aging and age-related disease: From mechanisms to therapy. Nat. Med..

[75] Coppé J.P., Patil C.K., Rodier F., Sun Y., Muñoz D.P., Goldstein J., Nelson P.S., Desprez P.Y., Campisi J. (2008). Senescence-associated secretory phenotypes reveal cell-nonautonomous functions of oncogenic RAS and the p53 tumor suppressor. PLoS Biol..

[76] Morin C.L., Pagliassotti M.J., Windmiller D., Eckel R.H. (1997). Adipose tissue-derived tumor necrosis factor-α activity is elevated in older rats. J. Gerontol. A Biol. Sci. Med. Sci..

[77] Starr M.E., Evers B.M., Saito H. (2009). Age-associated increase in cytokine production during systemic inflammation: Adipose tissue as a major source of IL-6. J. Gerontol. A Biol. Sci. Med. Sci..

[78] Maggio M., Guralnik J.M., Longo D.L., Ferrucci L. (2006). Interleukin-6 in aging and chronic disease: A magnificent pathway. J. Gerontol. A Biol. Sci. Med. Sci..

[79] Freund A., Orjalo A.V., Desprez P.Y., Campisi J. (2010). Inflammatory networks during cellular senescence: Causes and consequences. Trends Mol. Med..

[80] Polo S.E., Jackson S.P. (2011). Dynamics of DNA damage response proteins at DNA breaks: A focus on protein modifications. Genes Dev..

[81] Nicassio F., Corrado N., Vissers J.H., Areces L.B., Bergink S., Marteijn J.A., Geverts B., Houtsmuller A.B., Vermeulen W., di Fiore P.P. (2007). Human USP3 is a chromatin modifier required for S phase progression and genome stability. Curr. Biol..

[82] Lancini C., van den Berk P.C., Vissers J.H., Gargiulo G., Song J.Y., Hulsman D., Serresi M., Tanger E., Blom M., Vens C. (2014). Tight regulation of ubiquitin-mediated DNA damage response by USP3 preserves the functional integrity of hematopoietic stem cells. J. Exp. Med..

[83] Tooley C.E., Petkowski J.J., Muratore-Schroeder T.L., Balsbaugh J.L., Shabanowitz J., Sabat M., Minor W., Hunt D.F., Macara I.G. (2010). NRMT is an α-*N*-methyltransferase that methylates RCC1 and retinoblastoma protein. Nature.

[84] Cai Q., Fu L., Wang Z., Gan N., Dai X., Wang Y. (2014). α-*N*-methylation of damaged DNA-binding protein 2 (DDB2) and its function in nucleotide excision repair. J. Biol. Chem..

[85] Bonsignore L.A., Tooley J.G., Van Hoose P.M., Wang E., Cheng A., Cole M.P., Schaner Tooley C.E. (2015). NRMT1 knockout mice exhibit phenotypes associated with impaired DNA repair and premature aging. Mech. Ageing Dev..

[86] Fang E.F., Scheibye-Knudsen M., Brace L.E., Kassahun H., SenGupta T., Nilsen H., Mitchell J.R., Croteau D.L., Bohr V.A. (2014). Defective mitophagy in XPA via PARP-1 hyperactivation and NAD^+^/SIRT1 reduction. Cell.

[87] Scheibye-Knudsen M., Mitchell S.J., Fang E.F., Iyama T., Ward T., Wang J., Dunn C.A., Singh N., Veith S., Hasan-Olive M.M. (2014). A high-fat diet and NAD^+^ activate Sirt1 to rescue premature aging in cockayne syndrome. Cell Metab..

[88] Soskic V., Groebe K., Schrattenholz A. (2008). Nonenzymatic posttranslational protein modifications in ageing. Exp. Gerontol..

[89] Lee R.E., Brunette S., Puente L.G., Megeney L.A. (2010). Metacaspase Yca1 is required for clearance of insoluble protein aggregates. Proc. Natl. Acad. Sci. USA.

[90] Witko-Sarsat V., Nguyen-Khoa T., Jungers P., Drueke T.B., Descamps-Latscha B. (1999). Advanced oxidation protein products as a novel molecular basis of oxidative stress in uraemia. Nephrol. Dial. Transplant..

[91] Zhu Z., Chung W.H., Shim E.Y., Lee S.E., Ira G. (2008). Sgs1 helicase and two nucleases Dna2 and Exo1 resect DNA double-strand break ends. Cell.

[92] Böhm S., Mihalevic M.J., Casal M.A., Bernstein K.A. (2015). Disruption of SUMO-targeted ubiquitin ligases Slx5-Slx8/RNF4 alters RecQ-like helicase Sgs1/BLM localization in yeast and human cells. DNA Repair.

